# Oncogenetic Function and Prognostic Value of DNA Topoisomerase II Alpha in Human Malignances: A Pan-Cancer Analysis

**DOI:** 10.3389/fgene.2022.856692

**Published:** 2022-07-07

**Authors:** Fulai Zhao, Junli Chang, Peng Zhao, Wenyi Wang, Xingyuan Sun, Xiaoping Ma, Mengchen Yin, Yongjun Wang, Yanping Yang

**Affiliations:** ^1^ Longhua Hospital, Shanghai University of Traditional Chinese Medicine, Shanghai, China; ^2^ Key Laboratory of Theory and Therapy of Muscles and Bones, Ministry of Education, Shanghai, China

**Keywords:** *DNA topoisomerase II alpha*, pan-cancer analysis, oncogenesis, immune cell infiltration, genetic alteration, prognostic value

## Abstract

Increasing studies have revealed significant associations between *TOP2A* with oncogenesis and prognosis of human cancers; however, pan-cancer analysis has not been reported. Here, we explored the potential carcinogenic function and the association with clinical outcomes of *TOP2A* in 33 different human cancers. The results showed that *TOP2A* was amplified in 31 investigated cancers; *TOP2A* expression was significantly associated with metastasis of six different cancers and significantly associated with the survival of patients in ten different cancers; TOP2A-encoded protein was obviously upregulated in five available cancers; phosphorylated TOP2A protein at S1106 was significantly upregulated in all six available cancers. Moreover, *TOP2A* expression was found to be associated with the cancer-associated immune cell infiltration, including fibroblasts, Tregs, and macrophages. In addition, the Kyoto encyclopedia of genes and genomes (KEGG) pathway and Gene Ontology (GO) enrichment analyses revealed a most significant association between *TOP2A* with the Wnt signaling pathway and DNA conformation change. This work provides a comprehensive knowledge of *TOP2A* in different cancers, including carcinogenic function, prognostic values for metastasis, and clinical outcomes.

## Introduction

Based on the estimates of GOLBOCAN 2020, almost 19.3 million people were suffering from cancer and about 10.0 million deaths due to cancer were reported worldwide in 2020. Also, in 112 of 183 countries, cancer was reported to be the first or second leading cause of death for patients younger than 70 years. It is expected that there will be a 47% increase by 2040 with 28.4 million new cancer cases (excluding basal cell carcinoma) (Ferlay et al., 2018; ICGC/TCGA Pan-Cancer Analysis of Whole Genomes Consortium, 2020). Therefore, it is urgent to identify new biomarkers for the diagnosis and treatment of cancers.

Given the heterogeneity arises and complexity of carcinogenesis, it is very important to perform a pan-cancer analysis of a potential biomarker gene to promote the development of combination therapies and personalized medicine by evaluating its association with the clinical prognosis and involved molecular mechanisms (Hoadley et al., 2018).

**FIGURE 1:**
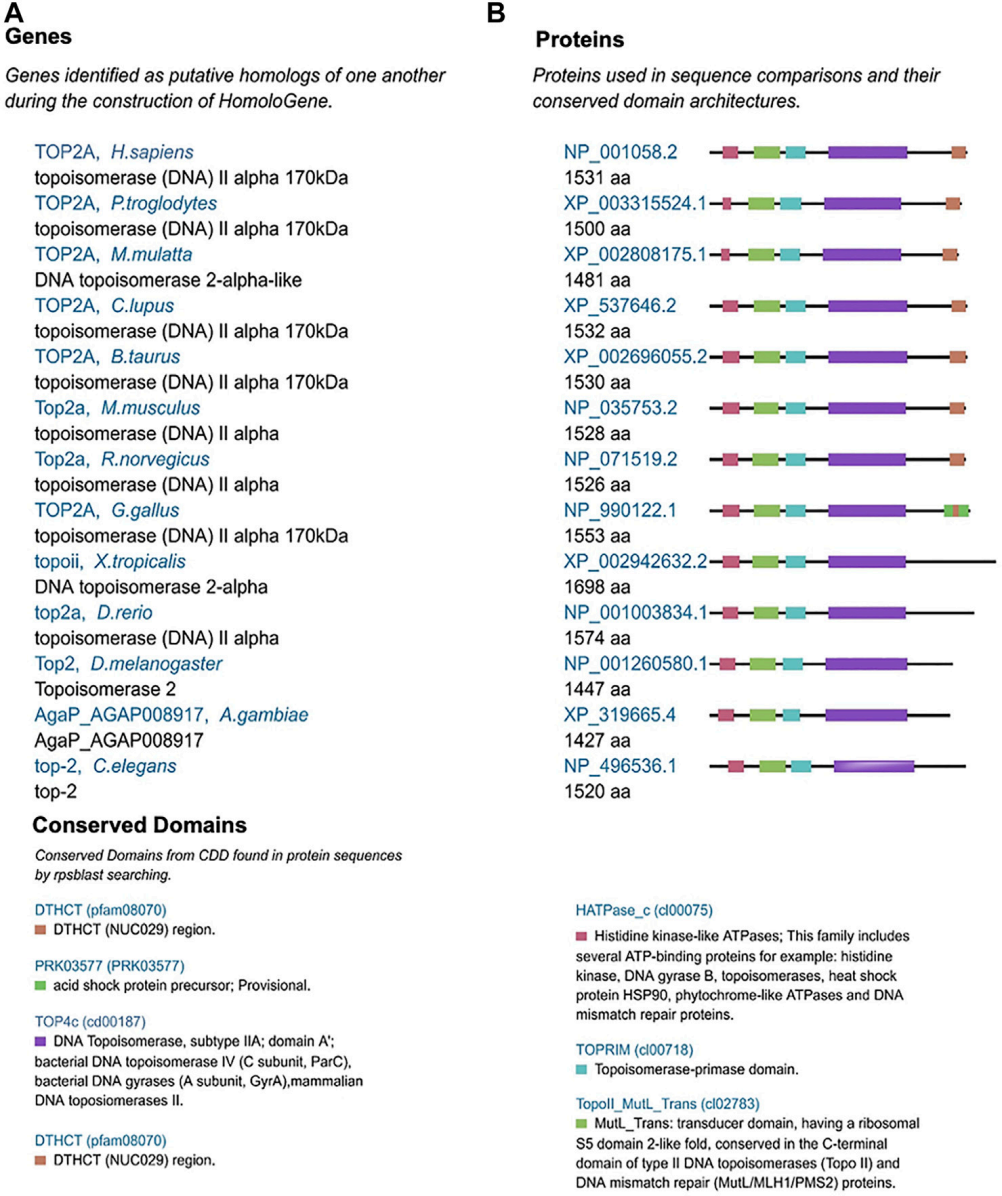
Conserved *TOP2A* **(A)** genes and **(B)** protein domains in different species.

DNA topoisomerase is a nuclear protein, which regulates the spatial dynamics of DNA and plays an important role in life activities, such as DNA replication, transcription, recombination, and chromosome separation (Champoux, 2001). Topoisomerase has two isoenzymes of topoisomerase I (*TOP1*) and topoisomerase II (*TOP2*), the latter includes two subtypes of *TOP2A* and *TOP2B* (McKinnon, 2016). *TOP2B* mainly exists in terminally differentiated cells (Isik et al., 2015), while *TOP2A* expression is cell-dependent and necessary for dividing cell survival (Cobb et al., 1997). The protein structure of *TOP2A* is conserved among different species (e.g., *H. sapiens*, *P. troglodytes*, and *M. mulatta*) with two common domains of DTHCT (pafam08070) and TOP4c (cd00187) (Figure 1), which regulates DNA topological state during transcription for chromosome condensation and chromatin separation by catalyzing the transient breaking and rejoining of two strands of duplex DNA. (Jain et al., 2013). Upregulated *TOP2A* has been reported in different cancers and is identified to be associated with poor prognosis (Depowski et al., 2000; Erguden et al., 2012; Jain et al., 2013; Parker et al., 2014). A long-term clinical cohort study showed that high expression of *TOP2A* in breast cancer patients can be used as a marker for the application of anthracycline-containing chemotherapeutics.(Leo et al., 2011). However, current research on *TOP2A* focus on single or limited cancer types. Therefore, it is time to explore the pan-cancer evidence of the biological functions of *TOP2A* cross-carcinoma to reveal the pathogenesis of *TOP2A* in different cancers or the potential molecular mechanisms in the clinical prognosis, so as to develop more specific treatments for various cancer types.

In the current study, a comprehensive pan-cancer analysis of *TOP2A* among 33 solid tumors was performed based on the public databases of TCGA and GEO, including the multiple factors of the gene/total protein expressions, the survival prognostic value, the genetic alteration, the protein mutation, the protein phosphorylation, the immune cell infiltration and the related signaling pathways of *TOP2A*.

## Methods and Materials

### Gene Expression Exploration

To identify the differently expressed *TOP2A* gene between various cancers/specific cancer subtypes and paired paracancerous normal tissues based on TCGA projects, “*TOP2A*” was used as the input term for the online “Gene_DE” module of TIMER2 (tumor immune estimation resource, version 2) (http://timer.cistrome.org/) (Li et al., 2020). For those cancers with extremely less or without paracancerous normal tissues [such as TCGA-ACC (Adrenocortical carcinoma) and TCGA-DLBC (Lymphoid Neoplasm Diffuse Large B-cell Lymphoma)], “Expression analysis-Box Plots” module of Gene Expression Profiling Interactive Analysis version 2 (GEPIA2) online tool (http://gepia2.cancer-pku.cn/#analysis) (Tang et al., 2019)was used to achieve the box plots of differently expressed genes between cancerous and corresponding normal tissues from the Genotype-Tissue Expression (GTEx) database based on the cutoff values: log2FC (fold change) = 1, *p*-value = 0.01, and “Match TCGA normal and GTEx data”. Moreover, the violin or box plots were obtained from log2 [TPM (Transcripts per million)+1] transformed expression data; meanwhile, the violin plots were also achieved from TNMplot (differential gene expression analysis in Tumor, Normal, and Metastatic tissues) online tool (http://www.tnmplot.com) (Bartha and Gyorffy, 2021) and the Gene-chip data, to discover *TOP2A* expression in tumor tissues, normal tissues, and metastatic tissues with the Kruskal–Wallis test for significance assay of Gene-chip data.

The UALCAN portal website (http://ualcan.path.uab.edu/analysis-prot.html) is used to analyze the mRNA expression of *TOP2A* in primary tissues and its association with clinical staging.

Protein expression investigation based on the CPTAC (Clinical proteomic tumor analysis consortium) dataset was performed using the online UALCAN portal, which provides an easy access to the publicly available cancer OMICS data (Chen et al., 2019). Therefore, we respectively identified the expressions of total or phosphorylated *TOP2A* (NP_001058.2) (with phosphorylation sites of S1106, S1374, S1213, S1247, S1351, S1354, S1377, S1393, S1525, S1374S1377, S1351S1354, and T1343S1351S1354) between primary cancer and normal tissues using “*TOP2A*” as input. Six cancer datasets were finally included in this study, including lung adenocarcinoma (LUAD), uterine corpus endometrial carcinoma (UCEC), renal cell carcinoma (clear cell RCC), breast cancer, ovarian cancer, and colon cancer.

### Prognosis Investigation

The GEPIA2 online “Survival Map” module (Tang et al., 2019) was applied to analyze disease-free survival (DFS) and overall survival (OS) significance map data of *TOP2A* among all cancers in the TCGA dataset. Survival curves were plotted by the GEPIA2 “Survival Analysis” module, using log-rank in hypothesis test and median expression values as cutoff of high- and low-expression cohorts.

### Genetic Alteration Assay

**FIGURE 2:**
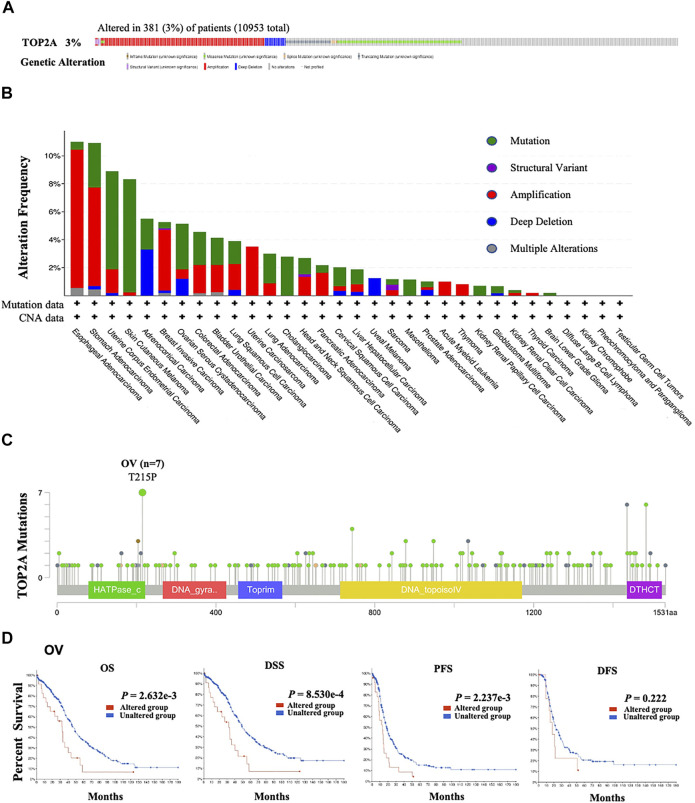
*TOP2A* mutations in all cancers from TCGA database. The cBioPortal tool was applied to analyze the mutation characteristics of *TOP2A* in the TCGA database, the **(A)** Genomic alteration frequencies and types; **(B)** genetic alteration frequencies (381/10953; 3%) and types of *TOP2A* gene in 10953 cancer patients with 10967 samples; **(C)** the alteration sites of TOP2A protein; **(D)** associations between the *TOP2A* alteration status of *TOP2A* with the overall survival, the disease-specific survival, the progression-free survival and the disease-free survival in OV patients were analyzed by the cBioPortal tool.

*TOP2A* genetic alteration features were analyzed using “*TOP2A*” as the querying term in the “Quick select” section of “TCGA Pan-Cancer Atlas Studies” in the cBioPortal online tool (https://www.cbioportal.org/) (Cerami et al., 2012; Gao et al., 2013). Mutation type, alteration frequency, and copy number alteration (CNA) across all cancers in the TCGA database were obtained based on the “Cancer Types Summary” module. Meanwhile, the information on the *TOP2A* genetic mutation site can be displayed by the “Mutations” module in the schematic diagram of the protein structure (Figure 2C). Then, based on the differences in overall survival, progression-free survival, and disease-free survival for cancer cases in the TCGA database without or with *TOP2A* genetic alteration were analyzed with the “Comparison” module. Kaplan-Meier method with log-rank *p*-value was used for plotting the survival curves.

### Investigation of Tumor-Immune Cell Infiltration

The online “Immune-Gene” module of TIMER2 was applied to investigate association between *TOP2A* gene expression and tumor-immune cell infiltrates across all cancers in the TCGA database, including cancer-associated fibroblasts, Tregs, and macrophages. The immune cell infiltration was evaluated with TIMER, CIBERSORT, CIBERSORT-ABS, QUANTISEQ, XCELL, MCPCOUNTER, and EPIC, the seven different algorithms. *p-* and partial correlation (cor)-values were calculated with purity-adjusted Spearman’s rank correlation test. Data visualization was achieved by heat maps and scatter plots.

### Enrichment Analysis of *TOP2A* Related Genes

A single protein name (“*TOP2A*”) AND an organism (“*Homo sapiens*”)were first queried online using STRING (https://string-db.org/) (Szklarczyk et al., 2019), followed by setting the following parameters of active interaction sources (“experiments”), max number of interactors to show (“no more than 50 interactors” in first shell), the meaning of network edges (“evidence”) and minimum required interaction score [“Low confidence (0.150)”] to collect the available TOP2A-binding proteins validated by experiments. Then the obtained experimentally confirmed TOP2A-binding proteins were further visualized using Cytoscape. Moreover, using the online bioinformatics tool “HIPLOT” (Yu et al., 2012) (https://hiplot.com.cn/), the Kyoto encyclopedia of genes and genomes (KEGG) pathway analysis, and the Gene Ontology (GO) enrichment analysis including cellular component, molecular function and biological process of two sets of data after being combined. Two-tailed *p <* 0.01 indicated statically significance.

## Results

Gene and total protein expressions of *TOP2A* in different malignancies, pathological stages, and metastatic status.

**FIGURE 3:**
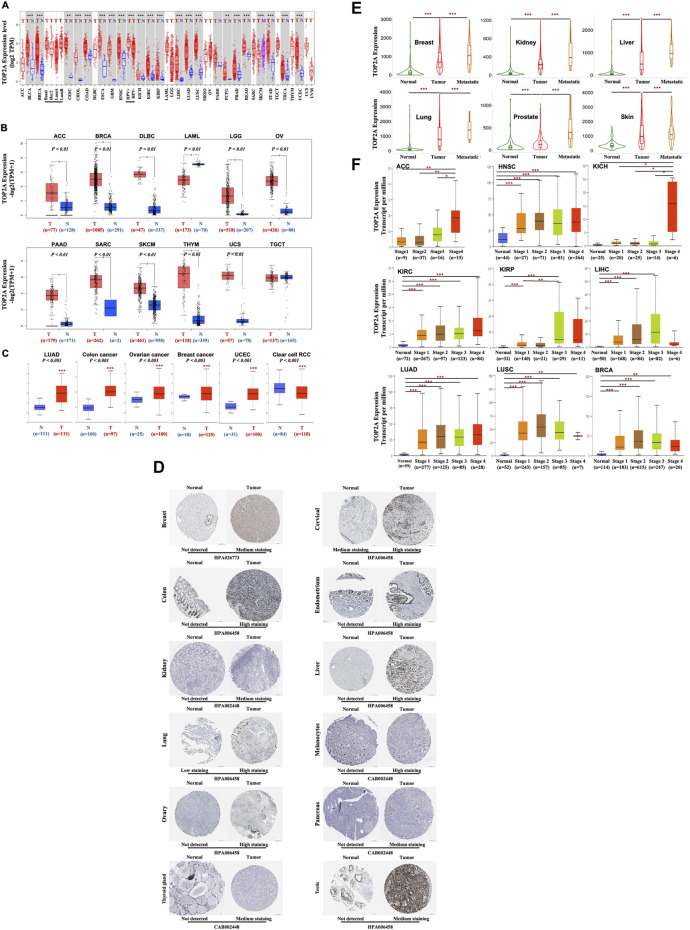
*TOP2A* gene and total protein expressions in different malignancies, pathological stages, and metastatic status. **(A)** Differently expressed *TOP2A* gene between various malignancies with/without specific malignant subtypes and the available normal control using the TIMER2 online tool based on TCGA database were presented as box plots **(B)** Differently expressed *TOP2A* gene between cancers of ACC, BRCA, DLBC, LAML, LGG, OV, PAAD, SARC, SKCM, THYM, UCS and TGCT from TCGA database and the available normal control from GTEx database were presented as box plots. *p < 0.01 **(C)** Differently expressed TOP2A total protein between primary cancer (LUAD, colon cancer, ovarian cancer, breast cancer, UCEC, and the clear cell RCC) and the paired normal tissues based on the CPTAC dataset. *** *p <* 0.001. **(D)** Representative immunohistochemistry images of TOP2A protein expressions in different cancer and normal control tissues from Human Protein Atlas **(E)** TNMplot for differently expressed *TOP2A* gene expressions in normal, cancerous, and metastatic cancerous tissues of breast, kidney, liver, lung, prostate, and skin **(F)** Boxplots showing *TOP2A* expression in stage-1, -2, -3 and -4 of ACC, BRCA, HNSC, KICH, KIRC, KIRP, LIHC, LUAD and LUSC with or without normal control based on TCGA database. *p < 0.05, ** *p <* 0.01; *** *p <* 0.001.

To investigate the oncogenic function of human *TOP2A*, the expression of *TOP2A* mRNA (NM_001067.4) between the cancerous and normal control tissues, or between the primary and metastasis cancer was analyzed using the TIMER2 online tool in 33 different cancers from the TCGA database. As shown in Figure 3A, *TOP2A* expression in tumor tissues was significantly increased versus the corresponding normal control tissues, including bladder urothelial carcinoma (BLCA), breast invasive carcinoma (BRCA), cholangiocarcinoma (CHOL), colon adenocarcinoma (COAD), esophageal carcinoma (ESCA), glioblastoma multiforme (GBM), head and neck squamous cell carcinoma (HNSC), kidney chromophobe (KICH), kidney renal clear cell carcinoma (KIRC), kidney renal papillary cell carcinoma (KIRP), liver hepatocellular carcinoma (LIHC), lung adenocarcinoma (LUAD), lung squamous cell carcinoma (LUSC), pancreatic adenocarcinoma (PADD), prostate adenocarcinoma (PRAD), rectum adenocarcinoma (READ), stomach adenocarcinoma (STAD), thyroid carcinoma (THCA), uterine corpus endometrial carcinoma (UCEC), cervical squamous cell carcinoma and endocervical adenocarcinoma (CESC), pheochromocytoma and paraganglioma (PCPG). Meanwhile, *TOP2A* mRNA expression was also found to be significantly increased in metastasis skin cutaneous melanoma than in the primary skin cutaneous melanoma.

Moreover, the expression of *TOP2A* mRNA between normal and tumor tissues for those cancers without data of normal control in the TCGA database was performed using the data of corresponding normal tissues from the GTEx dataset as normal controls. As shown in Figure 3B, *TOP2A* mRNA expression was significantly upregulated in tumors than in the normal control tissues, including adrenocortical carcinoma (ACC), lymphoid neoplasm diffuses large B-cell lymphoma (DLBC), brain lower-grade glioma (LGG), ovarian serous cystadenocarcinoma (OV), pancreatic adenocarcinoma (PAAD), sarcoma (SARC), skin cutaneous melanoma (SKCM), thymoma (THYM), uterine carcinosarcoma (UCS). However, *TOP2A* mRNA expression was downregulated in the acute myeloid leukemia (LAML) than in the normal control tissues and showed no significant difference between the testicular germ cell tumors (TGCT) and the normal control tissues.

To further evaluate the differences in TOP2A protein (NP_001058.2) expression between normal and tumor tissues, the protein expression patterns of *TOP2A* in cancer patients were first extracted from the Human Protein Atlas (HPA). As shown in Figure 3D, TOP2A protein was significantly upregulated in cancer than in normal control tissues, including breast, cervical, colon, endometrium, kidney, liver, lung, melanocytes, ovary, pancreas, thyroid gland, and testis, which were consistent with the expression panel of *TOP2A* mRNA in patients with same cancer based on TCGA database as shown in Figure 3B; meanwhile, the data from CPTAC dataset was analyzed using the online UALCAN portal, which also showed an increased expression of TOP2A protein in patients with primary breast invasive carcinoma, ovarian serous cystadenocarcinoma, colon adenocarcinoma, uterine corpus endometrial carcinoma, and lung adenocarcinoma than in normal tissues, and consistent with the expression panel of *TOP2A* mRNA in patients with same cancer; however, TOP2A protein showed a lower expression in kidney renal clear cell carcinoma (Figure 3C), which was inconsistent with the mRNA expression pattern in patients with kidney renal clear cell carcinoma based on TCGA database as shown in Figure 3A and protein expression pattern in patients with kidney renal clear cell carcinoma from the Human Protein Atlas (HPA) as shown in Figure 3D.

To analyze the association of *TOP2A* mRNA expression level with the metastatic abilities of different cancers, the “compare tumor, normal and metastasis” module of the TNMplot online tool was applied. The results showed that, in breast, kidney, liver, lung, prostate, and skin cancers, *TOP2A* expression was significantly upregulated in the tumor than in the normal control tissues; as well as in the metastatic versus the tumor tissues (Figure 3E).

To further explore the association of *TOP2A* mRNA expression level with the pathological stages of different cancers, the “Pathological Stage Plot” module of the Ualcan database (Figure 3F) was applied respectively, which revealed the highest mRNA expression of *TOP2A* in adrenocortical carcinoma, kidney chromophobe, kidney renal clear cell carcinoma, and lung adenocarcinoma appeared in stage 4, while in lung squamous cell carcinoma, head and neck squamous cell carcinoma and breast invasive carcinoma appeared in stage 3 (Figure 3F).

### Prognostic Value of *TOP2A* in Different Cancer Patients

**FIGURE 4:**
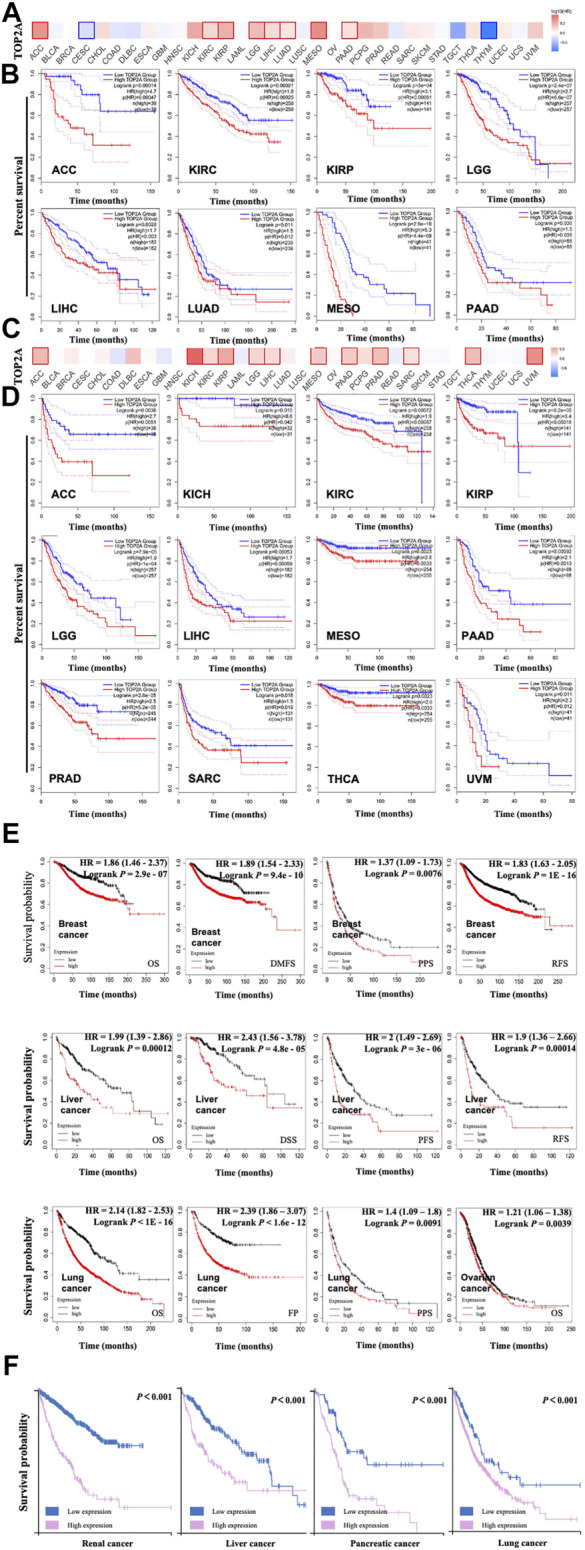
Prognostic association between *TOP2A* gene expression and survival of cancer patients based on TCGA database. GEPIA2 on line tool analyzed association between *TOP2A* gene expression with overall survival **(A, B)**, **(A)** Survival map of *TOP2A* gene with significant associations in ACC, KIRC, KIRP, LGG, LIHC, LUAD, MESO, and PAAD; **(B)** Survival curves of *TOP2A* gene with significant associations in ACC, CESC, KIRC, KIRP, LGG, LIHC, LUAD, MESO, PAAD, and THYM. GEPIA2 online tool analyzed the association between *TOP2A* gene expression with disease-free survival **(C, D)**, **(C)** Disease-free map of *TOP2A* gene with significant associations in ACC, KICH, KIRC, KIRP, LGG, LIHC, MESO, PAAD, PRAD, SARC, THCA, and UVM; **(D)** Disease-free survival curves of *TOP2A* gene with significant associations in ACC, KICH, KIRC, KIRP, LGG, LIHC, MESO, PAAD, PRAD, SARC, THCA, and UVM. **(E)** Correlation analysis between *TOP2A* gene expression and survivals (OS, DMFS, RFS, PPS, PFS, DSS, and FP) of cancer patients using Kaplan-Meier plotter. **(F)** The prognostic value of the *TOP2A* gene in renal cancer, liver cancer, pancreatic cancer, and lung cancer in the Human Protein Atlas dataset (HPA). High *TOP2A* group represents the high *TOP2A* expression group; the Low *TOP2A* group represents the low *TOP2A* expression group.

To explore the prognostic value of *TOP2A* expression level in different cancers, the cancer cases were divided into the high-expression group and the low-expression group according to the expression levels of *TOP2A* mRNA, followed by analyzing the associations between the *TOP2A* expression levels with the survival status of different cancer patients, based on the databases of TCGA and GEO, respectively. The results were visualized as a heat map (Figures 4A,C) and the survival curves with Kaplan–Meier method (Figures 4B,D,E,F). Our results identified that high *TOP2A* expression was significantly associated with the poor overall survival (OS) for cancers of adrenocortical carcinoma (*p =* 0.00014), kidney renal clear cell carcinoma (*p =* 0.00021), kidney renal papillary cell carcinoma (*p <* 0.001), brain lower grade glioma (*p <* 0.001), liver hepatocellular carcinoma (*p =* 0.0028), lung adenocarcinoma (*p =* 0.011), mesothelioma (*p <* 0.001), and pancreatic adenocarcinoma (*p =* 0.036) (Figure 4B), as well as significantly associated with the poor disease-free survival (DFS) for adrenocortical carcinoma (*p* = 0.0036), kidney chromophobe (*p =* 0.015), kidney renal clear cell carcinoma (*p =* 0.00072), kidney renal papillary cell carcinoma (*p <* 0.001), brain lower grade glioma (*p <* 0.001), liver hepatocellular carcinoma (*p =* 0.00053), mesothelioma (*p =* 0.011), pancreatic adenocarcinoma (*p =* 0.00092), prostate adenocarcinoma (*p <* 0.001), sarcoma (*p =* 0.018), thyroid carcinoma (*p =* 0.0023), uveal melanoma (*p =* 0.0016) based on TCGA database analyzed by GEPIA 2 online tool (Figure 4D).

To further confirm the prognostic value of *TOP2A* expression levels in different cancers, Kaplan–Meier method (https://kmplot.com/analysis/) (Nagy et al., 2021) was used to plot the survival curves, which presented a significant correlation between the high *TOP2A* expression and the poor survival status. As shown in Figure 4E, high *TOP2A* expression was found to be significantly associated with the poor OS (*p <* 2.9e-07), distant metastasis-free survival (DMFS) (*p =* 9.4e-10), post-progression survival (PPS) (*p* = 0.0076), and relapse-free survival (RFS) (*p <* 1E-16) of breast cancer patients; significantly associated with the poor OS (*p =* 0.00012), disease-specific survival (DSS) (*p =* 4.8e-05), progress-free survival (PFS) (*p =* 3e-06), and RFS (*p =* 0.00014) of liver cancer patients; significantly associated with the poor OS (*p <* 1E-16), first progression (FP) (*p =* 1.6e-12), and PPS (*p =* 0.0091) of lung cancer patients; as well as significantly associated with the poor OS of ovarian cancer (*p =* 0.039). And then “The Human Protein Atlas” module of “PATHOLOGY” was used to analyze the association between *TOP2A* expression level and the prognosis of different cancer patients, which demonstrated that the high *TOP2A* expression was closely related to the poor prognosis of renal, liver, pancreatic, and lung cancers (Figure 4F, all *p <* 0.001).

### Genetic and Protein Alteration Characteristic Analysis

To explore the influences of genetic and amino acid (AA) alterations on the carcinogenesis of different cancers, both the genetic alteration and AA mutation frequencies of *TOP2A* in all cancer patients from TCGA cohorts were analyzed using cBioportal For Cancer Genomics online tool. Our results showed that among the 10953 cancer patients, 381 cases appeared with *TOP2A* alteration with a frequency of 3% (381/10953), and amplification was the main genetic alteration type of *TOP2A* (Figures 2A,B). Furthermore, in *TOP2A*, the highest alteration frequency of “amplification” appeared in esophageal adenocarcinoma (>9%); of “mutation” appeared in skin cutaneous melanoma patients (>8%); and of “deep deletion” appeared in adrenocortical carcinoma (>3%). It is worth noting that uveal melanoma patients showed a ∼2% frequency of exclusive genetic alteration (deep deletion) in *TOP2A* (Figure 2B). As shown in Figure 2C, the 215 AA in the Tudor domain of TOP2A protein (T215P) showed the highest mutation frequency, which was detected in 7 ovarian serous cystadenocarcinoma cases.

Then, the potential association between *TOP2A* genetic alteration and the clinical survival prognosis of ovarian serous cystadenocarcinoma patients was analyzed based on these seven ovarian serous cystadenocarcinoma cases detected using the cBioportal For Cancer Genomics online tool. The ovarian serous cystadenocarcinoma patients with *TOP2A* genetic alteration showed worse prognoses in overall survival (*p* = 2.632e-03), disease-specific survival (*p* = 8.530e-03), and progression-free survival (*p =* 2.237e-03), but not disease-free survival (*p =* 0.222), versus those cases without *TOP2A* genetic alteration (Figure 2D).

### Protein Phosphorylation Analysis Data

**FIGURE 5:**
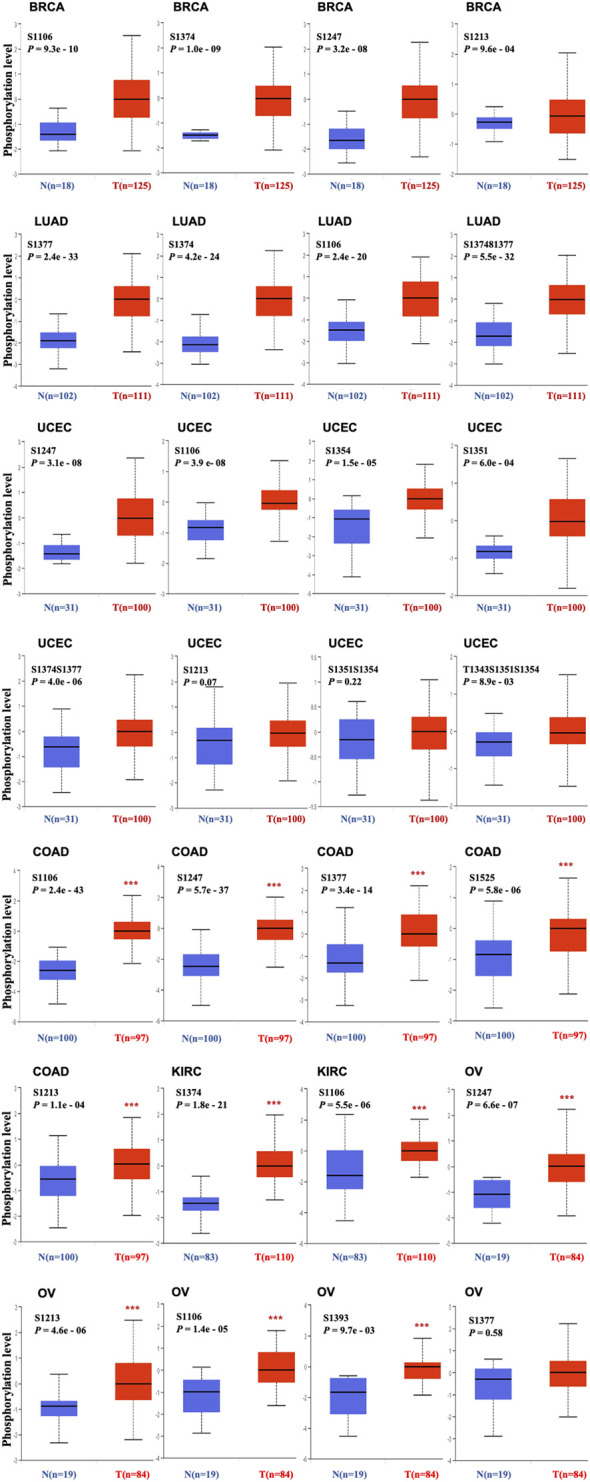
Phosphorylation level of TOP2A protein in various cancers. Expression levels of TOP2A protein with different phosphorylation sites (NP_001058.2, S1106, S1374, S1213, S1247, S1351, S1354, S1377, S1393, S1525, S1374S1377, S1351S1354 or T1343S1351S1354) between selected primary cancer and normal tissues analyzed by UALCAN on line tool based on the CPTAC dataset. Box plots for BRCA, LUAD, UCEC, COAD, KIRC, and OV.

To identify the differences in TOP2A phosphorylation levels with specific phosphorylation sites between normal tissues and primary tumor tissues, the CPTAC module in UALCAN web resource was applied for analysis over six different tumors (breast cancer, colon cancer, kidney renal clear cell carcinoma, lung adenocarcinoma, ovarian cancer, and uterine corpus endometrial carcinoma). The results revealed a higher S1106 phosphorylation level of TOP2A in all primary tumor tissues than in the normal tissues (Figures 5A–F).

### Immune Cell Infiltration Analysis Data

Given the importance of tumor-immune cell infiltrations in the initiation, development, and metastasis of cancers, the potential relationships between the *TOP2A* expression level with the tumor-immune cell infiltrations were analyzed in different estimations algorithms based on the TCGA database. The estimation algorithms of EPIC, MCPCOUNT, and TIED were used for the analysis of cancer-associated fibroblast infiltrates. The estimation algorithms of CIBERSORT, CIBERSORT-ABS, and QUANTISEQ were used for the analysis of Treg infiltrates. The estimation algorithms of EPIC, TIMER, and MCPCOUNT were used for the analysis of macrophage infiltrates. To further present the detailed information on the association between the *TOP2A* expression level with the tumor purity and the estimated infiltration value of tumor-immune cells, the results from one of the three estimation algorithms were provided.

**FIGURE 6:**
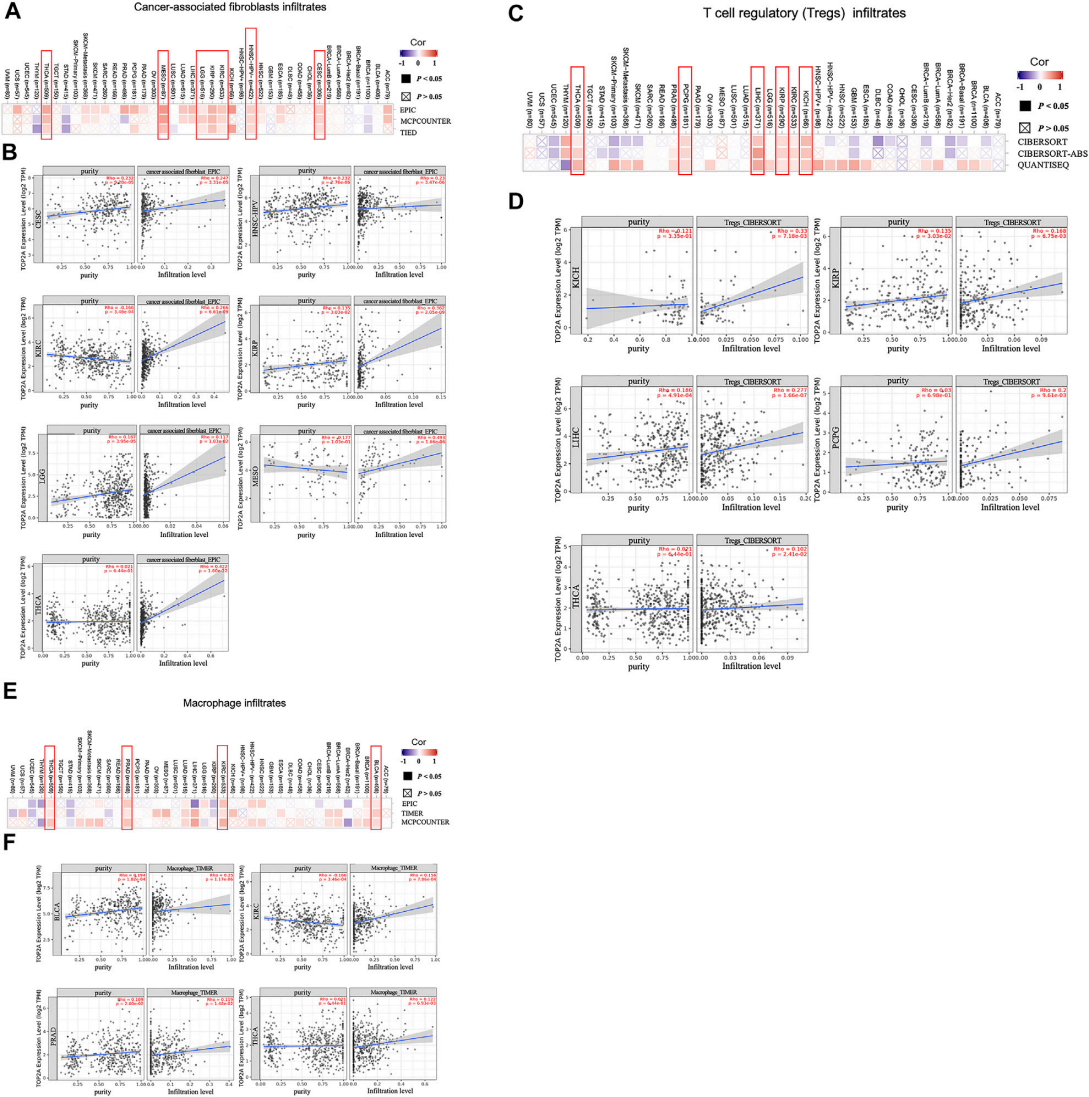
Association between *TOP2A* gene expression and tumor-immune cell infiltrates. **(A)** Correlations between the *TOP2A* expression with the abundance of cancer-associated fibroblasts in the estimation algorithms of EPIC, MCPCOUNT, and TIED; **(B)** associations of the *TOP2A* expression with the cancer-associated fibroblast infiltrates and the cancer purity; **(C)** correlations between the *TOP2A* expression with the abundance of Tregs in the estimation algorithms of CIBERSORT, CIBERSORT-ABS, and QUANTISEQ; **(D)**associations of the *TOP2A* expression with the Treg infiltrates and the cancer purity; **(E)** correlations between the *TOP2A* expression with the abundance of macrophage in the estimation algorithms of EPIC, TIMER and MCPCOUNT; **(F)**association of the *TOP2A* expression with macrophage infiltrates and the cancer purity.

The estimation algorithms of EPIC, MCPCOUNT and TIED together demonstrated a statistical positive correlation of *TOP2A* expression with the estimated infiltration value of cancer-associated fibroblasts in cervical squamous cell carcinoma and endocervical adenocarcinoma (CESC), human papillomavirus negative head and neck squamous cell carcinoma (HNSC-HPV), kidney renal clear cell carcinoma (KIRC), kidney renal papillary cell carcinoma (KIRP), brain lower-grade glioma (LGG), mesothelioma (MESO), as well as thyroid carcinoma (THCA) (Figure 6A); and the representative scatterplot data of the estimation algorithm of EPIC were provided, as shown in Figure 6B, the data revealed a statistically positive correlation between the *TOP2A* expression level with the cancer-associated fibroblast infiltrates and the cancer purity in the CESC, the HNSC-HPV, the KIRP, the LGG, and the THCA. However, *TOP2A* expression level showed a negative correlation with the tumor purity but a positive correlation with the estimated infiltration value of the cancer-associated fibroblasts in KIRC, while showed no correlation with the tumor purity but a positive correlation with the cancer-associated fibroblasts infiltrates in MESO.

Meanwhile, a statistical positive correlation of *TOP2A* expression level with the estimated infiltration value of Tregs in the kidney chromophobe (KICH), the kidney renal clear cell carcinoma (KIRP), the liver hepatocellular carcinoma (LIHC), the pheochromocytoma and paraganglioma (OCOG), and the thyroid carcinoma (THCA) were revealed by the estimation algorithms of EPIC, MCPCOUNT and TIED together (Figure 6C); and the representative scatterplot data of the algorithm of CIBERSORT were provided, as we can see in Figure 6D, the *TOP2A* expression level showed no statistical correlation with the tumor purity but a statistically positive correlation with the estimated infiltration value of Tregs in the PCPG, the KICH, and the THCA; showed a statistically positive correlation with the Treg infiltrates and the cancer purity in the KIRP and the LIHC.

The estimation algorithms of EPIC, TIMER, and MCPCOUNT together confirmed a statistical positive correlation between *TOP2A* expression and the estimated infiltration value of macrophages in the bladder urothelial carcinoma (BLCA), the kidney renal clear cell carcinoma (KIRC), the prostate adenocarcinoma (PRAD), and the thyroid carcinoma (THCA) (Figure 6E); and the representative scatterplot data of the algorithm of TIMER were provided, as we can see in Figure 6F, the *TOP2A* expression level showed no statistical correlation with the cancer purity, but a statistically positive correlation with the estimated infiltration value of macrophages in the THCA; showed a statistically positive correlation with the macrophage infiltrates and the cancer purity in the BLCA and the PRAD; and showed a negative correlation with the cancer purity but a statistically positive correlation with the estimated infiltration value of macrophages in the KIRC (Figure 6F).

### Enrichment Analysis of *TOP2A*-Related Partners

**FIGURE 7:**
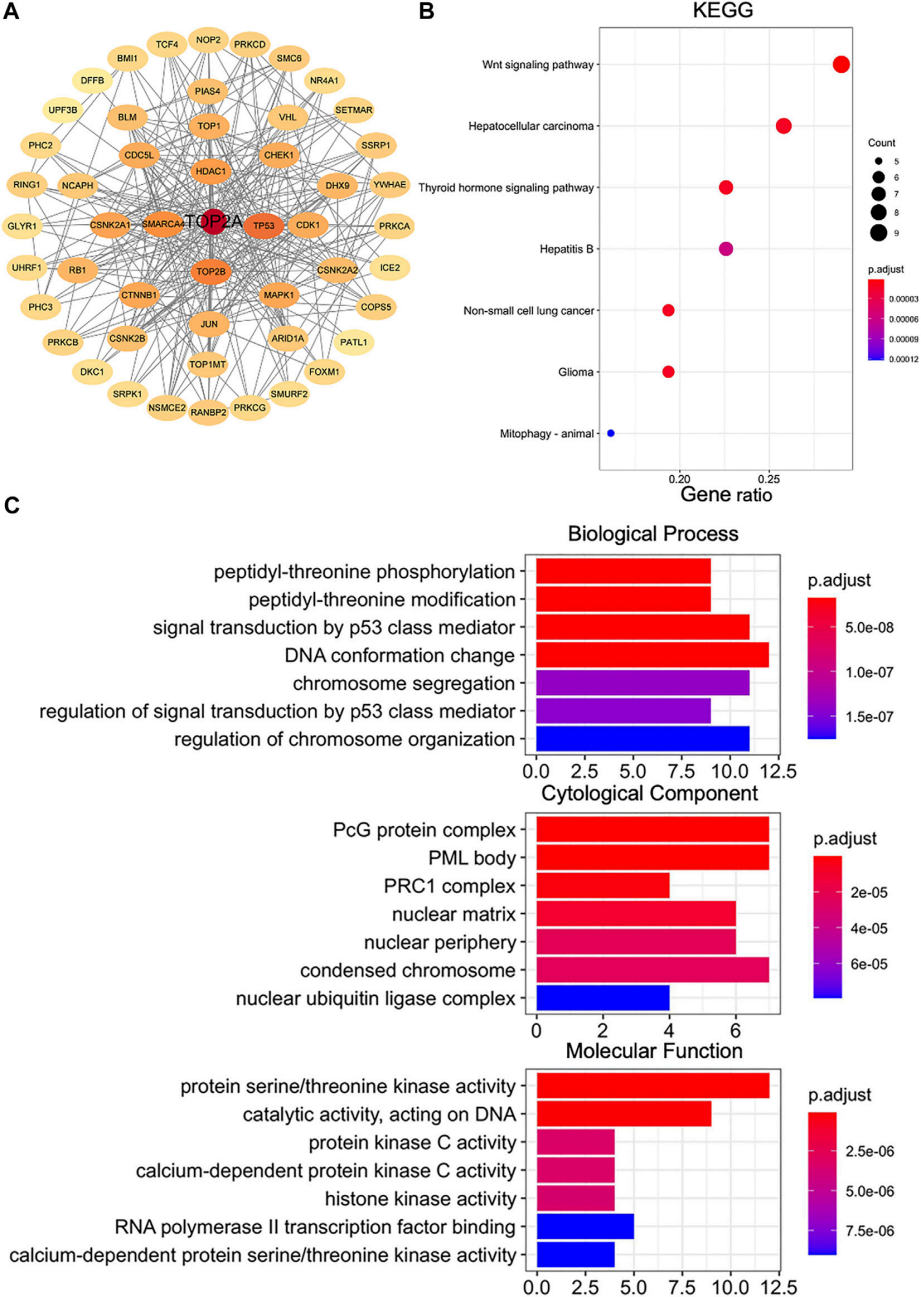
Enrichment analysis of *TOP2A*-related genes. Experimentally confirmed TOP2A-binding proteins obtained using the STRING online tool under the setting of “no more than 50 interactors,” **(A)** PPI network with Cytoscape; **(B)** KEGG pathway analysis; **(C)** Gene Ontology (GO) analysis.

To further investigate the involved molecular mechanisms of the *TOP2A* gene in tumorigenesis, the KEGG pathway and GO enrichment analyses were performed for the TOP2A-binding proteins. Based on the STRING online tool, 50 experimental validated TOP2A-binding proteins were identified and the protein-protein interaction was visualized with Cytoscape (Figure 7A). The KEGG pathway and GO enrichment analyses were then performed using the online bioinformatics tool “HIPLOT,” which showed that the “Wnt signaling pathway” might be the most important signaling pathway involved in *TOP2A* trigged tumor pathogenesis (Figure 7B); and the GO enrichment analysis data further indicated that most of these genes were linked to DNA structure, biological complex, and protein kinase activity, such as DNA conformation change, PcG protein complex, protein serine/threonine kinase activity (Figure 7C).

## Discussion

Increasing studies have revealed the importance of *TOP2A* in carcinogenesis, metastasis, and prognosis of different human cancers (Aumayr et al., 2018; Gao et al., 2020; Kou et al., 2020; Scott et al., 2020). Pan-cancer analysis of *TOP2A*, in terms of association with the clinical prognosis and involved molecular mechanisms based on profiling data, is necessary to understand the tumorigenesis mechanisms and explore the potential diagnostic biomarkers and treatment targets in clinical practice.

Our results based on the TCGA database showed that *TOP2A* mRNA was amplified in 31 out of 33 available tumor tissues except for testicular germ cell tumors, which only showed an increased trend without statistical significance in the tumor than in the normal control tissues (Our results based on TCGA database showed that *TOP2A* mRNA was amplified in 31 out of 33 available tumor tissues except for testicular germ cell tumors). These findings were further confirmed by analyzing the *TOP2A* mRNA expression profile based on the GEO database using the TNMplot online tool, furthermore, our results showed that *TOP2A* mRNA expression was higher in the metastatic tissues than in the paired tumor tissues of breast cancer, kidney cancer, liver cancer, lung cancer, prostate cancer, and skin cancer tissues.

Moreover, high *TOP2A* mRNA expression was also significantly associated with poor overall survival and diseases free survival of patients with adrenocortical carcinoma, breast invasive carcinoma, kidney renal clear cell carcinoma, kidney renal papillary cell carcinoma, brain lower grade glioma, liver hepatocellular carcinoma, lung adenocarcinoma, mesothelioma, or pancreatic adenocarcinoma. Consistent with mRNA expression profile, TOP2A protein expression determined by immunohistochemistry assay was also significantly up-regulated in 12 available tumor tissues. These findings indicate that *TOP2A* can be used as an independent prognostic predictor of cancers mentioned above.

Studies have shown that genomic alterations, such as mutations, small nucleotide polymorphisms, translocations, deletions, and insertions, occur more often in tumors than in normal tissues, which may involve carcinogenesis (Vedham and Verma, 2015; Palangat et al., 2019). Our results identified a 3% mutation rate of *TOP2A* in all 10953 tested cancer patients, and *TOP2A* mutation was significantly associated with poor overall survival, disease-specific survival, and progression-free survival in patients with ovarian serous cystadenocarcinoma, while the associations between TOP2A mutation with survival prognosis of other cancers needs to be further investigated.

Protein phosphorylation is a post-translational modification, which is one of the most important regulatory mechanisms in cells (Zolnierowicz and Bollen, 2000). Phosphorylation of TOP2A-specific sites occurs in a cell cycle-dependent manner and also regulates the sensitivity to TOP2A targeted drugs (Chikamori et al., 2003). In this study, we used the CPTAC dataset to explore the molecular mechanisms of TOP2A protein in breast cancer, clear cell renal cell carcinoma, lung adenocarcinoma, ovarian cancer, and uterine corpus endometrial carcinoma in terms of total protein and phosphoprotein levels. Our findings revealed higher expressions of both total and S1106 phosphorylated TOP2A proteins in the primary tumors than in the normal control tissues, suggesting the importance of S1106 phosphorylated TOP2A protein in tumorigenesis and drug resistance, and the necessity of exploring S1106 phosphorylated TOP2A protein-related mechanisms to design new therapeutic strategies for cancers resistant to *TOP2A* targeted drugs.

Infiltration of different types of immune cells is closely associated with initiation, development, and metastasis of cancers, thus a promising source of novel diagnostic and prognostic biomarkers (Fridman et al., 2011; Steven and Seliger, 2018). Many challenges remain in the extensive application of tumor immunotherapy (Murciano-Goroff et al., 2020; Zhang and Zhang, 2020), therefore, it is important to fully understand the status of tumor-immune cell infiltration and explore new biomarkers for improving tumor immunotherapy. At present, the association between *TOP2A* expression and tumor-immune cell infiltration has not been fully reported. By using multiple immune deconvolution methods to detect the correlation between *TOP2A* expression and the infiltration levels of different immune cells, including cancer-associated fibroblasts, Tregs, and macrophages, in available cancers, we found out that *TOP2A* expression was significantly associated with tumor-immune cell infiltration, indicating that *TOP2A* expression can reflect the status of tumor-immune cell infiltration.

KEGG and GO enrichment analysis of *TOP2A* functional related genes can help to reveal their potential functions and involved molecular mechanisms. *TOP2A* expression has been confirmed to be significantly associated with the Wnt signaling pathway, thyroid hormone signaling pathway, and DNA conformation changes. The findings in our current study were consistent with the literature, which reported that *TOP2A* induced the malignant characteristics of tumors by activating the Wnt/β-catenin signaling pathway (Pei et al., 2018; Liu et al., 2021).

Though the pan-cancer analysis of *TOP2A* in our research has a certain significance, there are still some limitations in our research such as data resources and experimental proof. Firstly, the data sets were from the TCGA database, whose data can only be incompletely downloaded and is lack follow-up data. Therefore, in the future, more efforts will be put into the establishment of our own data sets through observational research. Secondly, further basic experiments and clinical trials are worth performing to validate our findings and promote clinical translation.

In conclusion, our pan-cancer analysis of *TOP2A* showed that *TOP2A* was highly expressed in most cancers, and significantly correlated with cancer development and patient survival prognosis. These findings provide a comprehensive and systematic understanding of *TOP2A* in tumorigenesis from the perspective of clinical tumor samples; a possible and promising diagnostic and prognostic biomarker and a therapeutic target for multiple cancers.

## Data Availability

Publicly available datasets were analyzed in this study. This data can be found here: https://portal.gdc.cancer.gov/.
